# Differential expression of extracellular matrix components in the Fallopian tubes throughout the menstrual cycle

**DOI:** 10.1186/1477-7827-10-56

**Published:** 2012-08-16

**Authors:** Patricia S Diaz, Paula A Solar, Natalia E Juica, Pedro A Orihuela, Hugo Cardenas, Myron Christodoulides, Renato Vargas, Luis A Velasquez

**Affiliations:** 1 Centro para el Desarrollo de la Nanociencia y Nanotecnología, Universidad de Santiago, Santiago, Chile; 2 Laboratorio de Inmunología de la Reproducción, Facultad de Química y Biología, Universidad de Santiago, Santiago, Chile; 3 Center for integrative medicine and innovative sciences (CIMIS), Facultad de Medicina, Universidad Andrés Bello, Santiago, Chile; 4 Neisseria Research Group, Sir Henry Wellcome Laboratories, Division of Infection, Inflammation and Immunity, University of Southampton Medical School, Southampton, SO16 6YD, England, UK; 5 Servicio de Ginecología y Obstetricia, Hospital San José, Santiago, Chile

**Keywords:** Extracellular matrix, Metalloproteinases, Fallopian tubes, Menstrual cycle

## Abstract

**Background:**

One of the unique characteristics of the female genital tract is the extensive tissue remodeling observed throughout the menstrual cycle. Multiple components of the extracellular matrix take part in this tissue rebuilding; however, the individual components involved have not been identified.

**Methods:**

In the present study, the expression of extracellular matrix proteins and selected matrix metalloproteinase (MMP) activities in Fallopian tubes (FT) throughout the menstrual cycle were examined by PCR array, immunocytochemistry, zymography and bioinformatics.

**Results:**

Of the eighty-four genes analyzed, eighty-three were expressed in the FT during at least one stage of the menstrual cycle. We observed a significant increase (>/=2-fold) in ADAMTS1, ADAMTS13, COL7A1, MMP3, MMP9, PECAM1, and THBS3 in the periovulatory phase compared to the follicular phase. Meanwhile, we observed a significant decrease (>/= 2-fold) in COL7A1, ICAM1, ITGA8, MMP16, MMP9, CLEC3B, SELE and TIMP2 in the lutheal phase compared to the periovulatory phase. Immunocytochemistry showed that MMP-3 and MMP-9 were localized in the endosalpinx during all phases of the menstrual cycle. Gelatin zymograms detected non-cycle-dependent protease activity.

**Conclusions:**

Several extracellular matrix components were regulated throughout the menstrual cycle in a cyclic pattern, suggesting a possible steroid regulation and a role in tissue remodeling and FT functions.

## Background

One of the unique characteristics of the female genital tract is its extracellular matrix (ECM) layer, which plays a key role in the regulation of reproductive function. The ECM determines cell migration, division and differentiation, and ultimately cell survival or death. The ECM is regulated by a complex network of paracrine mediators [1], including matrix metalloproteinases (MMPs) and their endogenous inhibitors, the tissue inhibitors of metalloproteinases (TIMPs). MMPs participate in the degradation of the ECM [2], and more than 30 MMPs have been described in vertebrates, of which 27 are present in humans [3]. The ECM was long considered to be a passive structure that provided anchoring and mechanical support to the cells, and the extracellular proteases were thought to be simple remodelers of the ECM [4]. However, it is now generally accepted that the ECM is an active structure that contains growth factors, binding proteins and other bio-molecules, as well as binding sites for cellular surface molecules that are exposed only after undergoing proteolysis. Thus, the proteases responsible for the renewal of the ECM contribute dynamically to cellular interactions [5,6] and also have complementary effects on the recruitment of lymphocytes [7] and the processing of cytokines into their active forms [8].

Because of their potential to modify tissue, MMPs are strictly regulated at multiple levels, including transcription and zymogen activation, by extracellular inhibitors, their localization (within or outside the cell) and internalization by endocytosis. MMPs are secreted as inactive zymogens, and once activated, several mechanisms can regulate MMP activity [9-11]**.** Four types of MMP inhibitors are present in the extracellular spaces and in body fluids. The most relevant of these inhibitors are the TIMPs, which are 20–30 kDa proteins that interact directly with the active site(s) of MMPs. At present, four TIMPs have been described, namely, TIMP-1, TIMP-2, TIMP-3 and TIMP-4, although all of the TIMPs can inhibit all MMPs, TIMP-1 preferentially inhibits MMP-9, and TIMP-2 preferentially inhibits MMP-2 [3,12]**.** TIMPs are widely expressed in all tissues, and they are regulated jointly with the MMPs in most cases. In addition to inhibiting MMPs, the TIMPs participate in cell differentiation functions; e.g., TIMP-1 stimulates proliferative activity in endothelial cells [13], TIMP-2 stimulates fibroblast growth [14], and TIMP-3 induces apoptosis in the cells of the vascular smooth muscle [15].

Metalloproteinases are involved in regulating many of the structural ECM changes that occur during reproductive processes, participating actively in ovulation, menstruation, decidualization, trophoblast implantation, fetal development, and cervical dilation during childbirth [10,16-19]. In addition, embryonic developmental events, are characterized by cell migration processes, changes in tissue morphology, and the extensive restructuring of the ECM, which correlate with high levels of MMPs [20-25]. Notably, the activity of ovarian and uterine MMPs is regulated by the sex steroids, growth factors and cytokines that regulate reproductive function. However, despite the fact that the FT are subject to the same type of paracrine regulation, the MMP system has not been described in this organ, and its regulation during the menstrual cycle is also not known, thus limiting our understanding of tubal physiology and pathology and our ability to manage tubal disfunction**.**

## Methods

### Tissue collection

#### Ethics

All protocols were approved by the ethics and biosafety committee of the *Servicio de Salud Metropolitano Norte* and were in accordance with the ethical standards recommended by the Helsinki Declaration (1975). The FTs were obtained only from women undergoing surgical sterilization for reasons unrelated to this study and written informed consent was obtained from each participant of this study. The tissues were collected in collaboration with the *Servicio de Ginecología y Obstetricia* of the *Hospital San José*. The patients were fertile, 25 to 45 years of age, and voluntarily requested surgical sterilization. The patients fulfilled all of the requirements for the surgical procedure, as well as all of the criteria for inclusion in the study. Exclusion criteria included having suffered a sexually transmitted infection, tubal disease, PID, endometriosis, having used hormonal contraceptive methods within three months before surgery and heavy use of alcohol, tobacco or drugs abuse. A blood sample was obtained during each procedure such that the cycle stage could be assessed using plasma levels of estradiol and progesterone together with menstrual history. The piece of FT removed by laparoscopy was always the ampular segments**.**

### Processing of Fallopian tubes sampled at different stages of the menstrual cycle

The FT were processed in the laboratory immediately following extirpation using our standard method [22] and subsequently maintained in Dulbecco's Modified Eagle Medium (DMEM) at 37°C, 5% (v/v) CO_2_. The FT explants (1 cm^2^) were maintained for 24 h in culture medium to stabilize them and to ensure the absence of contaminating microorganism(s).

### RNA preparation

Total RNA was extracted from samples with TRIZOL (Invitrogen, USA) according to the manufacturer’s protocol. Contaminating genomic DNA was removed by RNase-free DNase (QIAGEN, USA**).** RNA yield, purity and concentration were determined by spectrophotometry, and 1 μg of each RNA was run on a 1% agarose gel (Invitrogen, USA) to ensure RNA integrity. The RT^2^ first-strand kit (Qiagen, USA) was used to convert 1 μg of RNA to cDNA according to the manufacturer’s protocol.

### ECM and adhesion molecule quantitative PCR arrays

Screening for the expression of 84 cell adhesion-related genes and ECM molecules in FT in 12 samples (four in the follicular phase, four in the periovulatory phase and four in the luteal phase; n = 12, duplicate arrays *per* FT sample) was conducted using the Human ECM and Adhesion Molecules RT^2^ Profiler PCR array (SaBiosciences Corp., Frederick, MD, USA) according to the manufacturer’s instructions. In brief, cDNA was prepared from 1 μg total RNA using the RT^2^ first-strand kit. PCR amplification was conducted with an initial 10-min step at 95°C followed by 40 cycles of 95°C for 15 s and 60°C for 1 min. The fluorescent SYBR Green signal was detected immediately after the extension step of each cycle, and the cycle when the fluoresence reach 0.1, was recorded as the cycle threshold. Data were imported into an Excel database and analyzed using the comparative cycle threshold method. The raw data were normalized to B2M, HPRT1 and RPL13A.

### Immunocytochemistry

Immunocytochemistry in nine FT samples was performed using anti-human antibody MMP9 (1:100 dilution, A b D Serotec, USA) and anti-human MMP3 (1:250 dilution, A b D Serotec, USA), respectively. Human placenta has been used as a positive control for the detection of both antigens. For the negative control, the first antibody was omitted from the reaction. Briefly, tissue sections were cut in 5 mm pieces and fixed with 4% Paraformaldehyde for 2 hours. Pieces were deep in a saccharose gradient (5% - 20%), embedded in OCT and stored at −20°C. Fixed samples on slides were rehydrated and treated with 1,5% hydrogen peroxide in distilled water for 30 min to block endogenous peroxidase activity. Cells were then washed with Phosphate-buffered saline-Tween 0,1% (PBS-Tween) and incubated with non-immune block for 60 min. Primary antibody was applied and incubated at 4°C for 16 h, followed by Histostain®-SP kits (Invitrogen, USA) according to the manufacturer’s instructions. Tissue sections were counterstained with Harris hematoxylin (Sigma, USA), air-dried and mounted. Finally, the background was subtracted using the software Image J (NIH,USA).

### Detection of MMP-associated gelatinolytic activity by zymography

Briefly, 25 μg of protein (determined by Bradford) from nine FT explant was subjected to electrophoresis on acrylamide/bisacrylamide gels copolymerized with gelatin. The presence of the different MMPs was determined based on the corresponding molecular weights of the visualized proteolytic bands and compared with pre-stained molecular weight marker standards and the corresponding positive MMP controls (Sigma, USA). The presence of SDS within the gels also allows the latent forms of proMMP-2 and proMMP-9 to be visualized. Enzyme activities in the gel were quantified with respect to both the surface and the intensity of the bands using an image analysis program (Biosciences gel systems, USA) and expressed in arbitrary densitometric units relative to the control (value of 1) MMP standard (Sigma, USA).

### Bioinformatics

The set of genes identified was analyzed using Gene Network Pro (Bioscience, USA), to identify cellular interactions previously described in other cell types.

### Statistical analyses

Data are expressed as the mean ± SD cycle threshold (Ct) for each sample and gene, and these data was used to calculated fold change in each phase. Student’s *t*-test was used when comparing two phases; follicular v/s periovulatory and periovulatory v/s lutheal, p-value <0.05 was considered significant.

## Results

### ECM and adhesion molecules quantitative PCR arrays

Of the 84 genes analyzed, 83 were expressed during some phase of the menstrual cycle, See additional file 1: Table S1.

**Figure 1:**
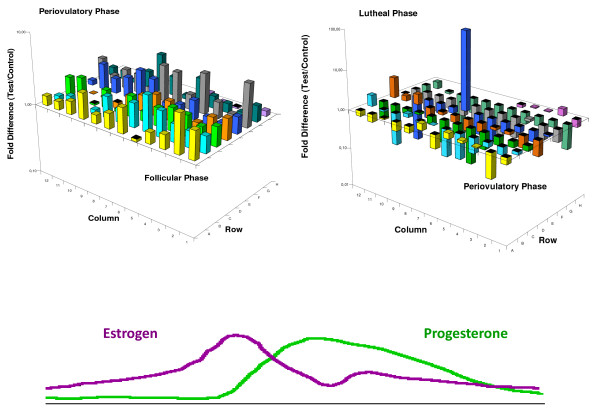
** Three-dimensional graph representing the gene expression levels in the PCR array plate.** The axes x and y are the coordinates that identify the genes and the Z axis represents the individual gene expression levels after comparing the follicular phase versus periovulatory phase (panel 1A) and the periovulatory phase versus the luteal phase (panel 1B). In both cases B2M, HPRT1 and RPL13A were use as normalizing agents. The color lines represent the estradiol and progesterone levels throughout the menstrual cycle.

Figure 1A shows the gene expression profile in the follicular phase versus the periovulatory phase when estradiol is the dominant hormone, and Figure 1B shows the periovulatory phase *versus* the luteal phase when progesterone is the dominant hormone. The expression profile of the genes studied shows that these genes tend to increase their expression in the periovulatory phase and decrease their expression in the luteal phase. These cyclical fluctuations in gene expression suggest that the expression of these genes is regulated by estradiol and progesterone.

When comparing the gene expression levels in the follicular phase versus the periovulatory phase, we found statistically significant differences in the expression of 7 genes (i.e., a ≥2-fold change). The p value and the fold change of each gene are detailed in Table 1.

**Table 1:** Genes with significant changes in gene expression between the follicular and periovulatory phases

**Symbol**	**P-value**	**Fold regulations**	**Molecular Class**
ADAMTS1	0.018	2.47	Metalloproteinases
ADAMTS13	0.044	3.52	Metalloproteinases
COL7A1	0.003	3.21	Structural collagen fibers
MMP3	0.014	4.01	Metalloproteinases
MMP9	0.036	3.60	Metalloproteinases
PECAM1	0.031	2.88	Cell adhesion protein
THBS3	0.009	2.28	Cell adhesion protein

When comparing the gene expression levels in the periovulatory phase versus the luteal phase, we found statistically significant differences in the expression of 8 genes. The p value and the fold change of each gene are detailed in Table 2.

**Table 2:** Genes with significant changes in expression between the periovulatory and luteal phases

**Symbol**	**P-value**	**Fold regulations**	**Molecular Class**
COL7A1	0.009	−4.24	Structural collagen fibers
ICAM1	0.049	−2.60	Glycoprotein
ITGA8	0.038	−2.91	Integrin, cell junction
MMP16	0.026	−14.32	Metalloproteinases
MMP9	0.008	−3.24	Metalloproteinases
CLEC3B	0.040	−11.19	Secreted polypeptide

Of the 8 genes with significant differences in gene expression levels during certain phase of the menstrual cycle, only COL7A1 and MMP9 showed significant differences in both phases of the menstrual cycle.

### Immunocytochemistry

**Figure 2:**
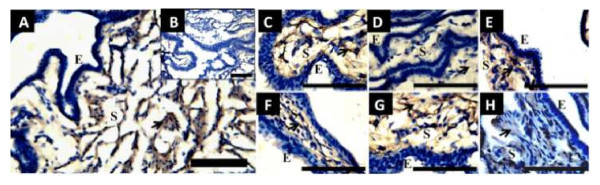
** Immunostainig of MMP-9 and MMP-3 in sections of human FT ampulla, throughout the menstrual cycle.** Positive staining of MMP-9 in follicular phase at lower magnification (**A**); note the complete absence of label in negative control in the small square at the right (**B**). Staining of MMP-9 at higher magnification in follicular (**C**), periovulatory phase (**D**) and luteal phase (**E**). Positive staining of MMP-9 in follicular phase (**F**), periovulatory phase (**G**) and luteal phase (**H**). Note the similar stromal distribution of MMP-9 and MMP-3 in the sections. The arrows indicates positive brown immunostaining, counterstaining was performed with hematoxylin. Scale bar = 100 μm.

Immunocytochemistry was used to localize the MMP3 and MMP9 proteins in histological FT sections in the follicular, periovulatory and luteal phases. All samples display a strong reaction in the endosalpinx, where the signal would be expected according to the functions performed by metalloproteinases in other tissues. The negative controls in which the primary antibody was omitted did not show any staining, indicating that the signal was specific (Figure 2).

### Detection of MMP-associated gelatinolytic activity by zymography

**Figure 3:**
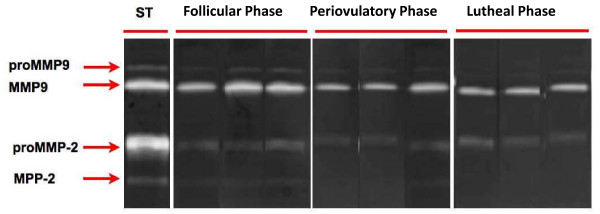
** Matrix metalloproteinase MMP-2 and MMP9 detected on FT on follicular, periovulatory and luteal phases.** MMP-2 and MMP-9 standard (Std) obtained from a commercial company. (**A**) Representative gelatin zymogram of three separate experiments illustrating pro and active MMP2 and MMP9 throughout the menstrual cycle. Data shows nine independent experiments. Each sample was assayed in duplicate.

Zymographic analyses were performed with selected samples of all phases of the menstrual cycle. As shown in Figure 3, all samples exhibited gelatinase activity. Densitometric analysis of Western blots showed that MMP2 and MMP9 were expressed at similar levels in all phases of the menstrual cycle (data not shown).

### Bioinformatics

**Figure 4:**
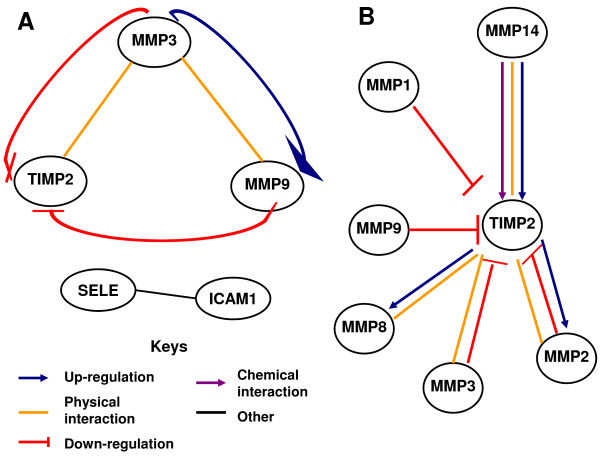
** Bioinformatic analysis.** Bioinformatic analysis of the genes identified to determine the potential cellular interactions between them (**A**). We also analyzed the potential regulatory pathways on TIMP-2, considering the variety and complexity of the interactions in which seems to be a crucial point in the cell regulation (**B**).

The set of genes identified was analyzed using Gene Network Pro to identify cellular interactions previously described in other cell types. The program detected several potential interactions between the identified genes, including physical interactions between MMP3/MMP9 [26] and between MMP3/TIMP-2 [27]; this analysis also suggested that MMP3 would be able to induce the activation of the MMP9 precursor. In turn, active MMP9 can inhibit TIMP-2 [27]. Finally, Selectin E and Cam1 participate in the NFkB pathway [28]. The potential interactions identified are detailed in Figure 4A. We also analyzed the potential regulatory pathways on TIMP-2, considering the variety and complexity of the interactions in which seems to be a crucial point in the cell regulation [27,29-32]. 

## Discussion

The main objective of this study was to determine how genes related to the extracellular matrix are expressed in the FT throughout the menstrual cycle. We used a PCR array plate that contained 84 genes selected for their role in extracellular matrix remodeling in various cell types.

An analysis of the overall behavior of this set of genes shows that 83 of the 84 genes are expressed in the FT during at least one phase of the menstrual cycle. These genes tend to be expressed at greater levels in the periovulatory phase than in the follicular phase and show reduced expression during the luteal phase. These results probably indicate that this set of genes is regulated by estradiol and progesterone.

Statistical analysis showed that 7 genes undergo a twofold increase in expression in the periovulatory phase compared to follicular phase. Of these genes, 1 encodes a structural protein (COL7A1), 4 encode genes that modify the extracellular matrix through proteolysis (ADAMTS1, ADAMTS13, MMP3, and MMP9) and two are involved in cell adhesion (PECAM1 and THBS3).

Briefly, ADAMTS1 (disintegrin and metalloproteinase with thrombospondin motif 1) has been associated with inflammatory processes, and its activity could be related to salpingitis triggered by sexually transmitted pathogens [33,34]. In addition, this gene is necessary for the development and functionality of the genital tract in rodents [35]. ADAMTS13 (ADAM metallopeptidase with thrombospondin type 1 motif 13) functions as a disintegrin and metalloproteinase with a thrombospondin motif. The enzyme encoded by this gene is the protease that cleaves the von Willebrand factor (vWF). Thus, this protein may participate in leukocyte migration that occurs in the fallopian tubes under both normal and pathological conditions. COL7A1 encodes the alpha chain of collagen type VII. The collagen fiber is expressed exclusively below the basal epithelial cells, and mutations in COL7A1 are associated with structural changes [36]. MMP3 is involved in the degradation of the extracellular matrix in several tissues [37] and is important for embryonic development, reproduction and tissue restructuring. MMP3 has also been associated with pathological processes [38], such as tumor metastasis and arthritis. PECAM1 (platelet/endothelial cell adhesion molecule 1) is expressed on the surfaces of platelets, neutrophils and T cells and at intercellular junctions in various cell types. This protein is a member of immunoglobulin superfamily and is involved in leukocyte migration, angiogenesis, and integrin activation [39]. THBS3 (thrombospondin 3) is an adhesive glycoprotein that is involved in cell-cell and cell-matrix interactions.

Eight genes exhibited a twofold or greater decrease in their expression in the luteal phase compared to the periovulatory phase. These genes encode proteins with structural functions, including proteolytic functions in the extracellular matrix (MMP16, MMP9, and TIMP2), three related to integrins and cell adhesion molecules (ICAM1, ITGA8, and SELE). COL7A1 is a structural protein, and CLEC3B participates in signal transduction process. Briefly, MMP16 activates MMP2 by cleavage in other cell systems. MMP9 (gelatinase B, 92 kDa gelatinase, 92 kDa type IV collagenase) degrades collagen IV and V. Murine studies suggest that it has a role in tissue remodeling associated with tumor development. TIMP2 is an inhibitor of matrix metalloproteinases and the only TIMP family member capable of suppressing the proliferation of endothelial cells. This protein is critical for maintaining tissue homeostasis by limiting tissue proliferation in response to angiogenic factors and inhibiting protease activity in tissues subjected to matrix remodeling. ICAM1 (intercellular adhesion molecule 1) is a cell surface glycoprotein that is typically expressed in endothelial cells and immune cells. This protein binds to CD11a/CD18- or CD11b/CD18-type integrins. SELE (E-selectin) is expressed on cytokine-stimulated endothelial cells and is believed to be responsible for the accumulation of leukocytes at sites of inflammation. Selectins are cell adhesion molecules that could be involved in the interaction between leukocytes and endothelium, as well as in the pathogenesis of atherosclerosis. CLEC3B (C-type lectin domain family 3, member B) is also downregulated in the luteal phase [40].

Using immunocytochemistry, we determined that MMP2 and MMP9 are expressed in the endosalpinx in a location consistent with a role in the remodeling of the fallopian tubes. Because MMPs are one of the principal regulators of the extracellular matrix, zymograms were performed at different stages of the menstrual cycle. These zymograms showed that MMP2 and MMP7 are expressed in the fallopian tube and that their enzymatic activity increases during the periovulatory phase, which is consistent with gene expression determined by PCR screen.

According to data from our bioinformatics analysis (Figure 4A), MMP9 and MMP3 negatively regulate TIMP2, suggesting that increasing the relative amount of MMP9 and MMP3 should decrease the relative amount of TIMP2, but this does not occur. Therefore, we analyzed the cellular interactions involved in the regulation of TIMP2 (Figure 4B) and analyzed the relative amounts of each gene. MMP14 and MMP2 showed the highest relative expression during different phases of the menstrual cycle in the initial PCR screen. In other cell systems, MMP14 increases the expression of TIMP2, which could explain the increase in TIMP2 during the periovulatory phase and its subsequent decrease in the luteal phase.

Like all reproductive tissue, the extracellular matrix of the FT should be remodeled during the luteal phase. Therefore, we analyzed the expression of genes related to matrix remodeling in other cell models. Several metalloproteinases were found to maintain their expression through the menstrual cycle, as shown in Figure 3. However, there was a decrease in the expression of MMP inhibitors (TIMPs) during the luteal phase, which could be related to extracellular matrix remodeling in human fallopian tubes during the luteal phase. This piece of information will be important for the generation of new patterns of remodeling, the normal and pathological tubal morphology.

## Conclusions

The overall results suggest that proteins related to extracellular matrix remodeling could participate in the changes that occur in the fallopian tube throughout the menstrual cycle, and these proteins are probably regulated directly or indirectly by estradiol and progesterone of ovarian origin. The specific role of each of the molecules identified must be defined in future studies. The function of the genes identified fits perfectly in the normal and pathological physiology of the TF, however the specific role of each of the molecules identified must be defined in future studies.

## **Abbreviations**

ADAMTS: ADAM metallopeptidase with thrombospondin type 1 motif 13; CLEC3B: C-type lectin domain family 3, member B; COL7A1: Collagen type VII alpha 1; ECM: Extracellular matrix; FT: Fallopian tubes; ICAM-1: Intracellular adhesion molecule 1; ITGA8: Integrin alpha 8; MMPs: Metalloproteinases; PECAM: Platelet-endothelial cell adhesion molecule; PID: Pelvic inflammatory disease; SDS: Sodium dodecyl sulfate; THBS3: Thrombospondin 3; TIMPs: Tissue inhibitors Metalloproteinases; v WF: von Willebrand factor.

## Competing interests

The authors declare that they have no competing interests.

## Authors’ contribution

PSD realize the zymography assays. PAS developed the real time PCR, performed bioinformatics and statistical analysis, and participate manuscript writing. NEJ participate in real time PCR runnig and prepare the immunocytochemistrys. PAO participate in the manuscript discussion. HC choose and done the statistical analysis. MC design the experiments in special the zymography assays. RV performed the laparoscopic operations, collect the fallopian tubes and LAV conceived of the study, and participated in its design and coordination and helped to draft the manuscript. All authors read and approved the final manuscript.

## Supplementary Material

Additional file 1 ** Table S1. **Gene expression levels of 84 genes related with extracellular matrix and adhesion proteins. The expresión levels in the diferentes phases of the menstrual cycle were expressed using 2-ΔCT formula (Columns B to D). The fold of changes in each phase is indicated in the columns E, F, G and was calculated with the 2-ΔΔCT algorism. Click here for file
